# QligFEP: an automated workflow for small molecule free energy calculations in Q

**DOI:** 10.1186/s13321-019-0348-5

**Published:** 2019-04-02

**Authors:** Willem Jespers, Mauricio Esguerra, Johan Åqvist, Hugo Gutiérrez-de-Terán

**Affiliations:** 1 Department of Cell and Molecular Biology, Uppsala University, Uppsala, 75124 Sweden; 2 Science for Life Laboratory, Uppsala University, Uppsala, Sweden

**Keywords:** Free energy perturbation (FEP), Molecular dynamics (MD), Ligand binding, Application programming interface (API), Dual topology

## Abstract

The process of ligand binding to a biological target can be represented as the equilibrium between the relevant solvated and bound states of the ligand. This which is the basis of structure-based, rigorous methods such as the estimation of relative binding affinities by free energy perturbation (FEP). Despite the growing capacity of computing power and the development of more accurate force fields, a high throughput application of FEP is currently hampered due to the need, in the current schemes, of an expert user definition of the “alchemical” transformations between molecules in the series explored. Here, we present QligFEP, a solution to this problem using an automated workflow for FEP calculations based on a dual topology approach. In this scheme, the starting poses of each of the two ligands, for which the relative affinity is to be calculated, are explicitly present in the MD simulations associated with the (dual topology) FEP transformation, making the perturbation pathway between the two ligands univocal. We show that this generalized method can be applied to accurately estimate solvation free energies for amino acid sidechain mimics, as well as the binding affinity shifts due to the chemical changes typical of lead optimization processes. This is illustrated in a number of protein systems extracted from other FEP studies in the literature: inhibitors of CDK2 kinase and a series of A_2A_ adenosine G protein-coupled receptor antagonists, where the results obtained with QligFEP are in excellent agreement with experimental data. In addition, our protocol allows for scaffold hopping perturbations to identify the binding affinities between different core scaffolds, which we illustrate with a series of Chk1 kinase inhibitors. QligFEP is implemented in the open-source MD package Q, and works with the most common family of force fields: OPLS, CHARMM and AMBER.

## Introduction

Calculating physicochemical properties of drug like molecules, such as the solvation free energies or the binding affinities for biological targets, has been a longstanding challenge to computational chemists. The recent increase in computational power and the improvement of force fields to accurately represent the physical properties of drug-like molecules [1–6] have set the grounds for molecular dynamics (MD) based methods to be routinely used to address these issues. Particularly, the rigorous free energy perturbation (FEP) methodology, despite being implemented in the framework of biochemical simulations decades ago [7], has recently gained recognition in the optimization of chemical modulators of biological targets, both in academia and pharmaceutical industry pipelines [8]. Besides the technical advances underlying the success of this approach, automatization tools are key to turn an otherwise time-consuming and error prone process into a systematically applicable tool [9, 10].

In contrast to plain, unbiased MD simulations, FEP simulations drive the conversion (or perturbation) of one molecule into another. If we think of two drug-like molecules for which the binding affinity for a given protein is compared, the FEP simulation will connect them through a series of unphysical intermediates [11]. If we translate this to the more typical exploration of a congeneric series of ligands around a given lead molecule, this can be seen as a system of nodes interconnected by edges, each of them representing a perturbation between the pair of molecules involved. If the collection of nodes is finite, e.g. in the case of amino acid mutations, one can define perturbation libraries for all possible permutations in the 20 × 20 matrix, and systematically apply this to evaluate ligand binding affinity for single-point protein mutants [12–14]. However, for drug like molecules the number of nodes considerable increases and the chemical space covered becomes several orders of magnitude larger (in the order of 10^33^ for all molecules adhering to Lipinski’s rule of five [15, 16]), making it impossible to predesign perturbation libraries for ligands. This introduces a bottleneck in the application of FEP simulations in real drug design projects, as the manual setup required is tedious, time consuming and prone to errors. This problem has been recognized by both the academia and industry and recently some protocols were proposed for a more efficient, automated setup of FEP simulations [9, 10, 17–19]. The underlying algorithms in those implementations are based on the calculation of maximum common substructure (MCS) between molecule pairs, and perturbations are performed along the edges of those connected nodes that preserve maximum similarity. Additionally, the selection of nodes can a posteriori be refined by a cycle closure analysis, which allows assessing the statistical errors by considering a given node more than once [17].

Next, a decision needs to be made on how to represent the edges, i.e. the perturbation pathway connecting the two molecules. Two major approaches have been proposed, single or dual topology [20]. A single topology consists of a one-to-one atom mapping between the two end point molecular systems. Here non-equivalent atoms in either of the end-states are represented by dummy particles (without non-bonded interactions) and only one set of coordinates need to be dealt with. This approach is particularly useful when changes between the molecules are small. However, breaking (or making) bonds becomes necessary when the bond topology changes more drastically (e.g. the opening of a ring structure in a molecule), which has been shown to significantly hamper convergence [21]. In such cases, a dual topology representation can be adopted. Here, the molecular entities from both nodes are simultaneously represented by their own set of Cartesian coordinates. In each end state of the simulation one of the molecules has its full interactions turned on, whereas the other is turned off (all dummy atoms). Since the total potential energy is a linear combination of the potential energy of each of the two states over the whole perturbation, the two molecules do not interact and the surrounding system sees a mixture of these two states [9, 22].

In this work, we describe an automated protocol to setup ligand perturbations utilizing a dual topology approach called QligFEP, which is implemented to interact with the open-source MD package Q. This software is specifically tailored to perform different types of free energy simulations, such as linear interaction energy (LIE), empirical valence bond (EVB) and FEP [23, 24]. Simulations are run in an explicit spherical droplet of water around the area of interest, i.e. the binding site. For free energy calculations, this has various advantages as compared to the more commonly used periodic boundary conditions (which are also implemented in Q), such as: (1) the absence of artificial periodicity in the finite system; (2) the possibility to calculate all interactions within the droplet accurately either directly or by utilizing multipole expansions for long-range interactions [23, 25]; (3) Focusing on the binding site whilst ‘cutting out’ the envelope allows to perform many multiple independent simulations, thus increasing the precision of the calculated free energies since the motions distal from the region of interest can be neglected. In fact, we have previously shown on the A_2A_ adenosine G protein-coupled receptor (GPCR) that such an approach increases convergence and accuracy compared to larger systems [13] as long as local structural fluctuations of the active site are sufficiently sampled [26, 27].

To illustrate the applicability of QligFEP and assess the validity of the most commonly used parameters, as well as advising on how to change those, we tested our workflow on various systems previously used to benchmark other FEP protocols. This includes calculations of solvation free energies of side chain mimics [28], which represent a diverse chemical space in terms of size, polarity and atomic constitution. Thereafter, we apply this methodology to calculate relative binding free energies of 16 Cyclin-dependent kinase 2 (CDK2) ligands, and compare the performance of three different force field families (OPLS, CHARMM and AMBER) to previously published work [29]. Next, relative binding free energies of a series of antagonists for the adenosine A_2A_ receptor are calculated and compared to a recently published approach [30, 31]. Finally, we show how the QligFEP dual-topology scheme can be applied for ‘scaffold hopping’, in the case of modifications of five Chk1 ligands of different chemotypes [32]. Our code and benchmark sets are available through GitHub (https://github.com/qusers/qligfep).

## Materials and methods

### Description of the workflow

**Fig. 1:**
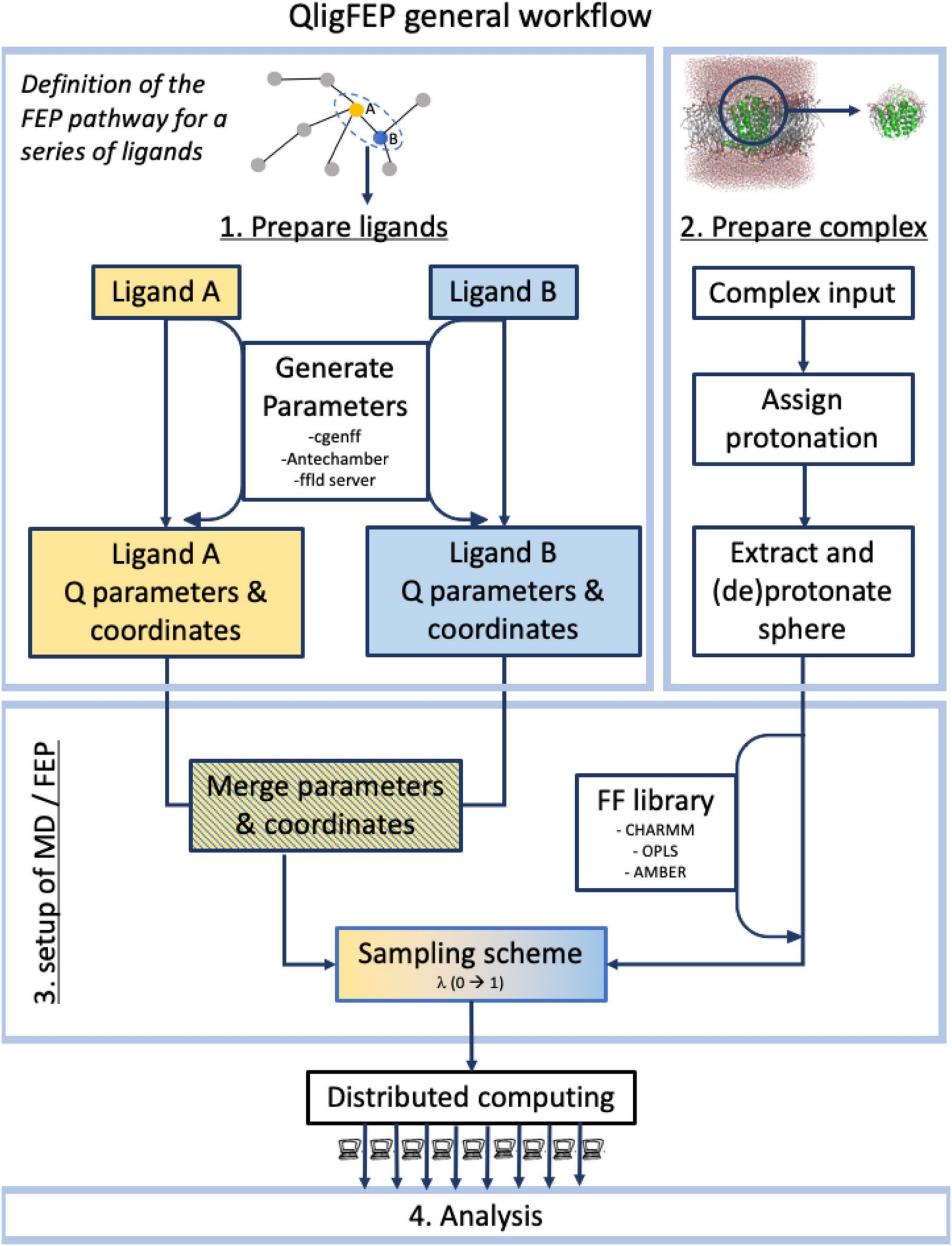
General workflow of the QligFEP API, which consists of three preparation steps and one analysis module. The user only needs to provide .pdb coordinate files for the first two steps, from where parameter and library files are generated to perform the FEP calculations. Currently, the CHARMM, AMBER and OPLS forcefields are supported

QligFEP is an application programming interface (API) that aims to automate and generalize the tedious process of setting up and analyzing the MD simulations for FEP calculations. Given a collection of molecules, for which we want to compare their relative binding affinities, a preliminary step involves the definition of the pair(s) of ligands to be compared. In QligFEP this can be done either manually, or by generating the pair list with an external program, e.g. LoMap [10], as represented in the top layer in Fig. 1. A good initial guess (adopted along this manuscript) is to define a radial pathway connecting all compounds to a central node (i.e. a single reference ligand). In cases where convergence is insufficient due to too big changes (assessed by a large SEM or even MD simulation crashes), one can add edges to nodes that involve smaller topological changes, as illustrated here in case of the A_2A_AR ligand binding calculations. The QligFEP workflow is then iteratively applied for each pair of molecules (A and B) in the pair list, and is composed of four modules (Fig. 1): (1) ligand parameterization, (2) complex preparation, (3) generation of the FEP and MD inputfiles and (4) an analysis tool.

#### Ligand parameterization

In the first module, the user provides PDB files of all small molecules that are going to be analyzed via FEP calculations. The coordinates of the molecules will be the input actually processed, i.e. this module does not enumerate protonation, enantiomeric and tautomeric states. The main aim of the module is thus to translate ligand 3D coordinates into Q readable library and parameter files, currently available for three families of force fields: OPLS-AA/M [33], Amber ff14sb [34] and CHARMM36 [3]. We provide a translation based on qtools [35] of the GAFF/AnteChamber [1, 36] parameters for AMBER. For CHARMM, the parameters are obtained and translated into Q format using CGenFF [37]. Our implementation of the OPLS force field for ligands includes the OPLS2005 version generated via Schrodinger’s ffld_server [38]. Note that one needs to obtain the required licenses for these external programs separately (which in some cases are free for academics). As an open source solution, LigParGen [5] can be used to generate Q readable parameter and library files based on the OPLS force field [5] and we are currently working to include other alternatives for ligand parameterization in the near future related e.g. to the OpenForceField consortium [39].

#### Complex preparation

In the second module, the macromolecular target is prepared for MD simulations of the binding site under spherical boundary conditions (SBC), as discussed in the introduction. The input is a PDB file, with or without hydrogens. In the former case the user should indicate which program has been used to add hydrogens, to account for the specific atom naming. If the module should, on the other hand, protonate the protein, the protonation state of ionizable residues is inferred from the residue name (e.g. HID, HIE, HIP for histidine protonated in delta, epsilon or positively charged, etc.), in accordance with the reference Q library files for proteins. This module also accounts for the two main parameters related to the implementation of the spherical boundary conditions in Q [40]: (1) the center of the sphere, which is normally placed at the center of geometry of a reference ligand, can be defined in different ways i.e. Cartesian coordinates, protein residue or a ligand atom, and (2) the size of the sphere, which should have a radius big enough such that it encompasses all the residues within the binding site, including a solvation patch to ensure sufficient dielectrical screening (e.g. 10–15 Å from the most distal atom in the ligand to the sphere boundary) [40]. The system is solvated with a pre-generated water grid in Q [23], and waters overlapping with either of the two ligands are automatically removed in the next stage. This module also accounts for the necessary neutralization of ionizable residues in the restrained area and outside the sphere dimensions [40].

Finally, this module also replicates the setup for the analogous calculations of the reference states for the FEP simulation, e.g. ligands in water for binding affinity calculations (or ligands in vacuum for the estimation of solvation free energies, see below).

#### Generation of the FEP and MD inputfiles

The third step prepares input files for the FEP simulations, according to a number of variables that should be carefully considered by the user. Given a pair of ligands A and B, their parameters (generated from step 1) are merged with the corresponding general force field parameters in Q, while the corresponding library files are merged into a dual topology library file (Fig. 1). These files are used together with the corresponding coordinates of the two ligands in the corresponding state, i.e. bound, solvated or vacuum, as indicated above, to build the topology file in each case. The subsequent FEP simulation, for which details are given below, will be guided by a number of input parameters, which are also generated at this stage and will define the desired sampling scheme. The FEP transformation involves sampling of a series of intermediate states along a pathway between the potentials, *U*_A_ and *U*_B_, corresponding to each of the molecules. The probability density between the configurational distributions of the two states must overlap sufficiently. Therefore, the pathway is divided in a series of discrete steps following a linear combination of the potentials according to $U_{i} = \left( {1 - \lambda_{i} } \right)U_{A} + \lambda_{i} U_{B}$, where the coupling parameter λ_t_ varies between 0 and 1, and an ensemble average is collected at each of these steps [41, 42]. It follows that the number of λ steps, their distribution along the FEP pathway (e.g. linear or non-linear), the sampling time per λ step, as well as the starting point (e.g., λ = 0, 0.5 or 1) and directionality of the simulations, are key parameters of the FEP simulation, which are defined in these modules. The simulations are setup as a number of replicate MD simulations (with different random initial velocities), and due to the convenience of using a distributed computing scheme (Fig. 1), we include the option to write out input files tailored for a specific (high performance) computing cluster.

#### Analyzing the simulations

The last module in QligFEP performs all required analysis to report free energies and the associated statistical parameters. This includes up to three different methods to calculate relative free energies: (1) Zwanzig’s exponential formula [43], (2) overlap sampling (OS) [44] and (3) Bennet’s acceptance ratio (BAR) [45], the latter method being the one reported throughout this manuscript. Standard errors of the mean (SEM) are estimated from the individual replicate simulations, The module provides a linear-regression statistical analysis (i.e. for retrospective analysis when experimental affinity values are available) including the Pearson R^2^ with associated 95% confidence intervals (CI, p = 0.05, based on the application of Fisher’s z transformation for application to small datasets [46]), and the mean absolute error (MAE), calculated using scikit-learn [47]. Further details as to the specifications of the command line input can be found in the manual, which includes several tutorials.

### System preparation

Prior to entering the workflow, protein and ligand coordinates must be generated and/or retrieved from the corresponding databases. We herein describe the details of this procedure for the dataset treated in this manuscript.

Ligand coordinates were generated in a reasonable 3D conformation with the appropriate tools in Maestro [48]. The starting point differed between the different test cases, i.e., drawing from scratch (the side-chain mimics), modifying the crystal coordinates of the reference ligand (scaffold hopping) or the docked poses kindly provided by Wang et al. (CDK2 inhibitors) [49], or those obtained by manual docking (A_2A_AR antagonists). Each molecule was subsequently treated as the input for the corresponding parameterization tools, managed by the “Ligand preparation” module as described above.

All protein structures were pre-processed using Protein Preperation Wizard in Maestro [50]. In the case of the A_2A_AR, the receptor was embedded in a lipid bilayer and shortly equilibrated using the protocols in the GPCR-ModSim web server (http://www.gpcr-modsim.org) as described elsewhere [13, 51–53]. Selected water molecules in the binding site (from the crystal structures of CDK2 and Chk1, or from the or the MD equilibration in the case of A_2A_AR) were initially retained, and the complex given as input for the “Complex preparation” module. In each case, the center of the sphere was automatically generated from the center of geometry of the two ligands involved in the perturbation, and the sphere radius was initially set to 25 Å, which is a good guess in most cases considering the typical size of drug-like molecules [40]. However, in the case of CDK2 inhibitors the radius was optimized to 22 Å as a combination of two factors: the relatively small size of the ligands in the dataset, and the fact that this smaller radius allowed to exclude from the simulated sphere a highly charged cluster of residues formed by Arg50, Arg126, Arg150 and a phosphorylated Thr160, which would otherwise not have enough solvation patch as they would lie on the sphere boundary [40].

### MD/FEP simulations

The software package Q [23] was used for the MD sampling under SBC with the following settings: atoms lying outside the simulation sphere were tightly constrained to their initial coordinates (200 kcal/mol/Å^2^ force constant) and excluded from the calculation of the non-bonded interactions. In the boundary of the sphere, solvent atoms subject to polarization and radial restrains using the surface constrained all-atom solvent (SCAAS) [23, 54] model to mimic the properties of bulk water at the sphere surface. Long range electrostatics interactions beyond a 10 Å cut off were treated with the local reaction field method [55], except for the atoms undergoing the FEP transformation where no cutoff was applied. Solvent bonds and angles were constrained using the SHAKE algorithm [56].

The system was then subjected to 10 parallel MD replicate simulations, which only differed in their initially assigned random (Maxwell-Boltzman) velocities. Each simulation includes an equilibration scheme as follows, which is the default in QligFEP: (1) an initial phase of 31 ps where the simulation sphere is heated from 0.1 to 298 K and a positional restraint of 25 kcal/mol/Å^2^ initially imposed on all solute heavy atoms slowly released; (2) a 100 ps unbiased and unrestrained equilibration. The subsequent production phase followed, where the sampling along a λ transformation pathway (defining the FEP transformation) was performed with the following parameters in the different datasets (unless indicated the contrary): the transformation was divided into 51 λ steps, evenly distributed (i.e. linear sampling), the corresponding MD sampling for each λ step being 10 ps using a 1 fs time step. Thus, each FEP transformation involved a total simulation time of 10 × (131 ps + (51 × 10 ps)) = 6.41 ns for each of the two legs in the thermodynamic cycle, resulting in a total sampling time of 12.82 ns per perturbation. For comparison, a typical perturbation in the commercial alternative FEP+ includes one simulation of 12 lambda windows, with 5 ns sampling per window giving 120 ns under a bigger system using PBC [30]. However, in cases where large changes are considered (i.e. more than 6 heavy atoms) the sampling above might be insufficient to achieve the desired convergence, which might be easily diagnosed by a high SEM or even simulation crashes. In such cases, one can increase the phase-space overlap by increasing the λ windows, or either using a more dense (non-linear) sampling around the initial/ending states, or just increase the sampling of each λ window to increase convergence. In this work, a different scheme was used for the larger perturbations involved in the datasets of the relative hydration free energies and the A_2A_AR antagonist binding.

In the dual topology paradigm, each perturbation involves parallel growing (A) or annihilation (B) of the relative atoms of the two ligands A and B, and includes the perturbation to/from soft-core potentials as an intermediate step to ensure sufficient convergence [57]. Half-harmonic distance restraints of 2.0 kcal/mol/Å^2^ were applied to maintain pairs of equivalent (non-dummy) atoms between the two ligands (A and B), within a window distance of 0.0–0.2 Å. Since this restraint is only applied on atom pairs, of which one of them will have the interactions fully turned off in each of the end states, the energetic term of the restraints cancels and no additional correction needs to be applied. The relative free energy (∆∆*G*_A-B_) is calculated by closing the corresponding thermodynamic cycle, where the ∆*G* corresponding to each of the two legs was calculated with the BAR approach [45]. When the ligand mutation involves a change in the total charge of the sphere, a Born correction term was added to the calculated free energies to account for the polarization effect [58], estimated as:

1 $$\Delta G_{Born} = - 332\frac{{Q_{I}^{2} }}{{2r_{Born} }}\left( {1 - \frac{1}{\varepsilon }} \right)$$

where *Q*_*I*_ is the net charge of the solute, and *r*_*Born*_ is the radius of the cavity in the macroscopic medium, with a dielectric constant *ε*, in which the charge is embedded.

All calculations were performed using the OPLS-AA/M [33] force field with TIP3P waters, except for the CDK2 inhibitor set where results are reported with the three families of force fields implemented in QligFEP. Standard errors of the mean (SEM) are estimated from the individual replicate simulations by QligFEP, whereas errors reported for FEP+ are either BAR analytical error estimations or bootstrap estimated errors as reported in their original publications [49].

## Results and discussion

In this section, we use the workflow described above in four different case scenarios, where the calculation of relative free energies can be correlated with experimental hydration or binding free energies for different series of molecules as reported in the literature. The first case accounts for estimation of solvation free energies, tested on the Wolfenden set of protein side chain mimics [28]. Next, QligFEP is used to automatically estimate relative binding affinities of ligand series in two different target systems: (1) a globular enzyme, cyclin-dependent kinase 2-cyclin A receptor (CDK2), where relative affinities of a dataset of 16 inhibitors are also used to evaluate the accuracy of the three different force fields implemented, and (2) antagonist binding to a G-protein coupled receptor, the adenosine receptor A_2A_ (A_2A_AR). Finally, we show how our perturbation protocol can accurately assist ‘scaffold hopping’ [59] modifications on a series of five Checkpoint kinase 1 (Chk1) inhibitors.

### Relative hydration free energies

The solubility of small molecules is a key physicochemical property of any drug candidate [60]. Therefore, there is much interest in accurate computational estimations of ligand solubility via e.g. calculation of their corresponding solvation free energies. FEP has been quite successfully applied to obtain such estimations, although force field and water models can significantly influence accuracy [61]. To calculate the water solvation (hydration) free energies, a thermodynamic cycle can be constructed where the calculation legs refer to perturbations of the molecules of interest in vacuum and water, respectively. Our test case consists of mimics of amino acid side chains, for which absolute hydration free energies have been well characterized [28]. Notably, these experimental values were determined in a way that, in case of ionizable residues, only neutral states of the side chain mimic were considered. Thus, to be able to compare the solvation free energies of the corresponding charged species, the hydration free energy of the proton needs to be taken into account [62]. Here we use the values reported by Tissandier et al. [63], as adopted by Kelly et al. [64] and included in the Minnesota solvation database [65, 66]. For a more detailed discussion on the hydration free energy of the proton we refer to the work of Zhang et al. [62]. The total set thus includes 23 amino acid mimics (Table 1), encompassing diverse molecules in terms of physicochemical properties for which hydration free energies cover an energetic span of more than 80 kcal/mol. Taking the smallest methyl group sidechain in alanine as a reference, we calculated relative hydration free energies between any given side chain mimic (X) to methane (Me), and compared to the relative hydration free energies provided in Wolfenden et al. (note that, given this strategy, glycine was excluded) [28]. The dual topology framework allows us to perform the calculations in three alternative ways: sidechain annihilation (X → Me), sidechain growing (Me → X), both following a classical λ (1 → 0) FEP pathway, or alternatively starting from a mixture of both states (λ = 0.5) and propagating in each direction (1 ← λ → 0). As reported in Table 1, the simulations are well converged with a very low SEM (± 0.23 kcal/mol on average) over all replicate simulations in each of the three cases. The hysteresis, defined as the difference between the values obtained in the forward (annihilation) and backward (creation) directions, is also generally very low, with an average value of 0.47 kcal/mol. Notably, in the cases involving the largest growth (i.e. for p-cresol and 3-methylindole in the Me → X pathway), the default sampling with 51 λ windows was not sufficient to ensure convergence, as none of the simulations ran to completion. In these cases, a second round with 101 λ windows were needed and the corresponding values reported in Table 1. This is a first example indicating that low convergence may be a criterion for revising the FEP strategy (see below for A_2A_ antagonists). Regardless of the perturbation scheme applied, the calculated solvation free energies show an overall excellent agreement with experiment, with an almost perfect correlation in terms of R^2^. Given the large span of free energies, this value indeed masks the accumulated deviation from experiment, and the R^2^ determined on the separate charged/neutral groups is around 0.9 for all methods (see Table 1). While the calculated MAE over the whole dataset falls within a range of 1.46–1.76 kcal/mol, most of this error is caused by the higher deviations for ionizable molecules, with a smaller error below 1 kcal/mol in the best case for neutral sidechains. This is to be expected, as it is well known that the accuracy of force fields for these moieties is lower, and various corrections are needed to account for artifacts introduced by periodicity and long-range electrostatic cut-offs [62]. In our case, the latter does not apply since simulations are performed in a finite spherical droplet of water, where no cut-off is used for any atoms undergoing the alchemical transformation, allowing us to apply a simple correction to the calculated free energies of charged species using Born’s formula [58]. This yields an overall MAE of 3.67 kcal/mol for the five charged residues, which is considerably lower than those reported with the same force field by Zhang et al. [62], which range from 5.52 to 20.23 kcal/mol depending on the correction applied. It is also interesting to compare the results of QligFEP to other computational studies which, using a variety of sampling techniques and force fields, did not report any calculations on the charged species [67–70]. The MAE obtained in the study considering the neutral form of the aminoacid mimics is 1.71 kcal/mol, which is outperformed by our results (Table 1, considering only non-charged residues MAE = 0.95–1.24 kcal/mol). Indeed the lowest MAE (0.69 kcal/mol) was reported from the Sandler group excluding any form of Asp, Glu, Lys or Arg [67], which in our case would yield an even lower value of MAE = 0.53 kcal/mol.

**Table 1:** Experimental and calculated relative solvation free energies of side chain mimics [28]

Sidechain mimic	Exp	Solvation free energies (∆∆*G* (kcal/mol)) X → CH_4_				λ_0_ ← λ_0.5_ → λ_1_
		λ_0_ → λ_1_				
		X → Me	Me → X	Average	Hysteresis	
Propane	− 0.05	0.20 ± 0.08	− 0.19 ± 0.19	0.20	0.01	0.08 ± 0.12
Isobutane	− 0.34	− 0.27 ± 0.16	0.22 ± 0.09	− 0.25	0.05	− 0.04 ± 0.12
1-butane	− 0.21	0.02 ± 0.25	− 0.33 ± 0.11	0.16	0.35	− 0.06 ± 0.11
Ethanol	6.82	7.05 ± 0.15	− 6.97 ± 0.11	7.01	0.08	6.75 ± 0.14
Methanol	7.00	6.69 ± 0.07	− 6.69 ± 0.07	6.69	0	6.62 ± 0.08
Methanethiol	3.18	2.01 ± 0.05	− 1.61 ± 0.09	1.81	0.4	1.78 ± 0.05
methylsulfanylethane	3.42	2.68 ± 0.08	− 2.39 ± 0.37	2.54	0.29	2.06 ± 0.11
Acetamide	11.62	10.91 ± 0.16	− 10.89 ± 0.08	10.90	0.02	10.93 ± 0.10
Propionamide	11.32	11.02 ± 0.31	− 11.09 ± 0.14	11.06	0.07	11.29 ± 0.15
Toluene	2.70	2.56 ± 0.48	− 1.96 ± 0.63	2.26	0.6	2.66 ± 0.35
p-cresol	8.05	8.16 ± 0.44	− 7.20 ± 0.12^a^	7.68^a^	0.96^a^	7.74 ± 0.48
4-methylimidaziole (N_δ_H)	12.21^b^	9.86 ± 0.09	− 8.35 ± 0.96	9.11	1.51	10.22 ± 0.13
4-methylimidaziole (N_ε_H)	12.21^b^	11.13 ± 0.15	− 10.85 ± 0.17	10.99	0.28	10.68 ± 0.34
3-methylindole	7.82	6.25 ± 0.37	− 5.25 ± 0.13^a^	5.75^a^	1.00^a^	7.41 ± 0.52
n-Propylguanidine (N_2_H_3_)	12.86	16.43 ± 0.44	− 15.47 ± 0.53	15.95	0.96	16.99 ± 0.29
Acetic acid (COOH)	8.64	8.16 ± 0.08	− 7.84 ± 0.09	8.00	0.32	8.07 ± 0.11
Propionic acid (COOH)	8.41	11.44 ± 0.17	− 11.41 ± 0.15	11.43	0.03	11.98 ± 0.23
butan-1-amine (NH_2_)	6.32	5.52 ± 0.22	− 4.37 ± 0.34	4.95	1.15	4.88 ± 0.38
n-Propylguanidine (N_2_H_4_^+^)	69.15	68.17 ± 0.51	− 67.05 ± 0.35	67.61	1.13	67.33 ± 0.71
Acetic acid (COO^−^)	79.52	82.64 ± 0.10	− 82.21 ± 0.22	82.42	0.43	82.71 ± 0.07
Propionic acid (COO^−^)	78.04	82.28 ± 0.21	− 82.63 ± 0.31	82.45	0.35	82.37 ± 0.13
butan-1-amine (NH_3_^+^)	73.07	77.71 ± 0.48	− 78.49 ± 0.16	78.10	0.78	78.39 ± 0.22
4-methylimidaziole (N_δ,ε_H2^+^)	64.23	67.75 ± 0.19	− 67.77 ± 0.18	67.75	0.02	67.42 ± 0.20
*Statistical figures*						
All sidechains	R^2^	1.00	1.00	1.00		1.00
	[95% CI]	[0.99–1.00]	[0.99–1.00]	[0.99–1.00]		[0.99–1.00]
	MAE	1.46	1.76	1.60		1.58
	Slope	1.05	1.05	1.05		1.05
	Intercept	0.43	0.89	0.66		0.43
Neutral sidechains	R^2^	0.91	0.88	0.90		0.89
	[95% CI]	[0.77–0.97]	[0.71− 0.96]	[0.74− 0.96]		[0.74–0.96]
	MAE	0.95	1.24	1.08		1.03
	Slope	0.98	0.98	1.00		1.04
	Intercept	0.21	0.43	0.32		0.36
Charged sidechains	R^2^	0.92	0.86	0.89		0.89
	[95% CI]	[0.23–0.99]	[0.07–0.99]	[0.14–0.99]		[0.13–0.99]
	MAE	3.33	3.67	3.48		3.57
	Slope	1.11	1.31	1.12		1.16
	Intercept	5.21	6.71	5.94		8.50

^a^Relative free energies were calculated using sigmoidal sampling and λ = 101 windows

^b^The population distribution of a single proton N_ε_H:N_δ_H is approximately 80:20 [62]

While the complete annihilation of a sidechain seems to work well in all cases, we noted that the sidechain growth needs a smoother protocol to be implemented for bigger substituents (e.g. see Me to p-cresol or 3-methylindole). Thus, we have adopted the ‘middle’ scheme (1 ← λ = 0.5 → 0) as the standard protocol throughout the rest of this manuscript, since it forms the best trade-off between general applicability and accuracy. There are cases where the user might want to have more control over endpoints, introduce a smoother lambda spacing scheme, or calculate averages over two endpoint simulations, which is easily achieved using QligFEP.

### Ligand binding to CDK2

**Fig. 2:**
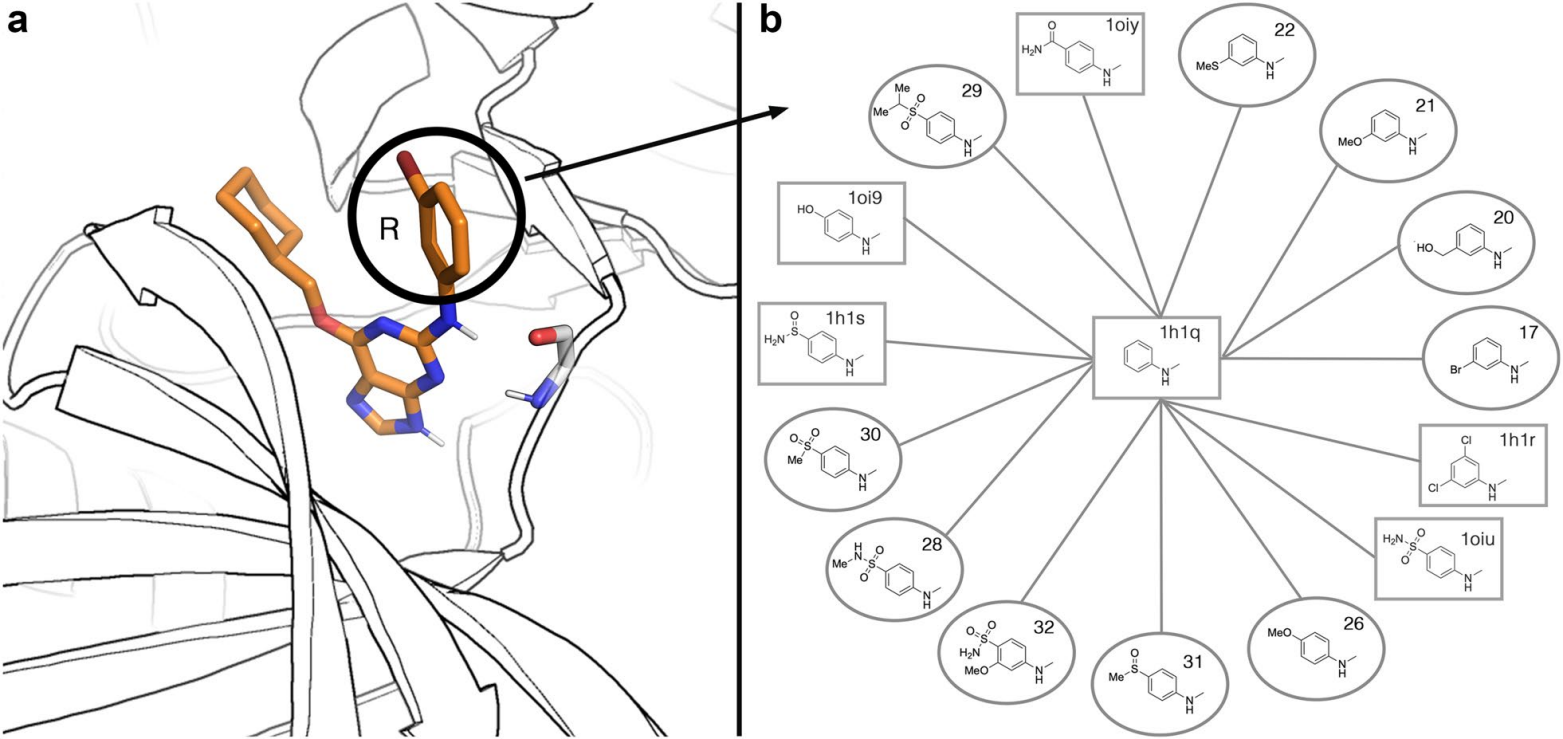
**a** Binding mode of CDK2 inhibitor **17**, showing key interactions with the backbone in the hinge region of the protein. All substituents introduced at R are positioned in a solvent exposed cavity on the surface on the protein. **b** Overview of the chemical constitution of the 16 R-groups (nodes) and selected perturbations (edges) for the calculations reported in Table 2

**Fig. 3:**
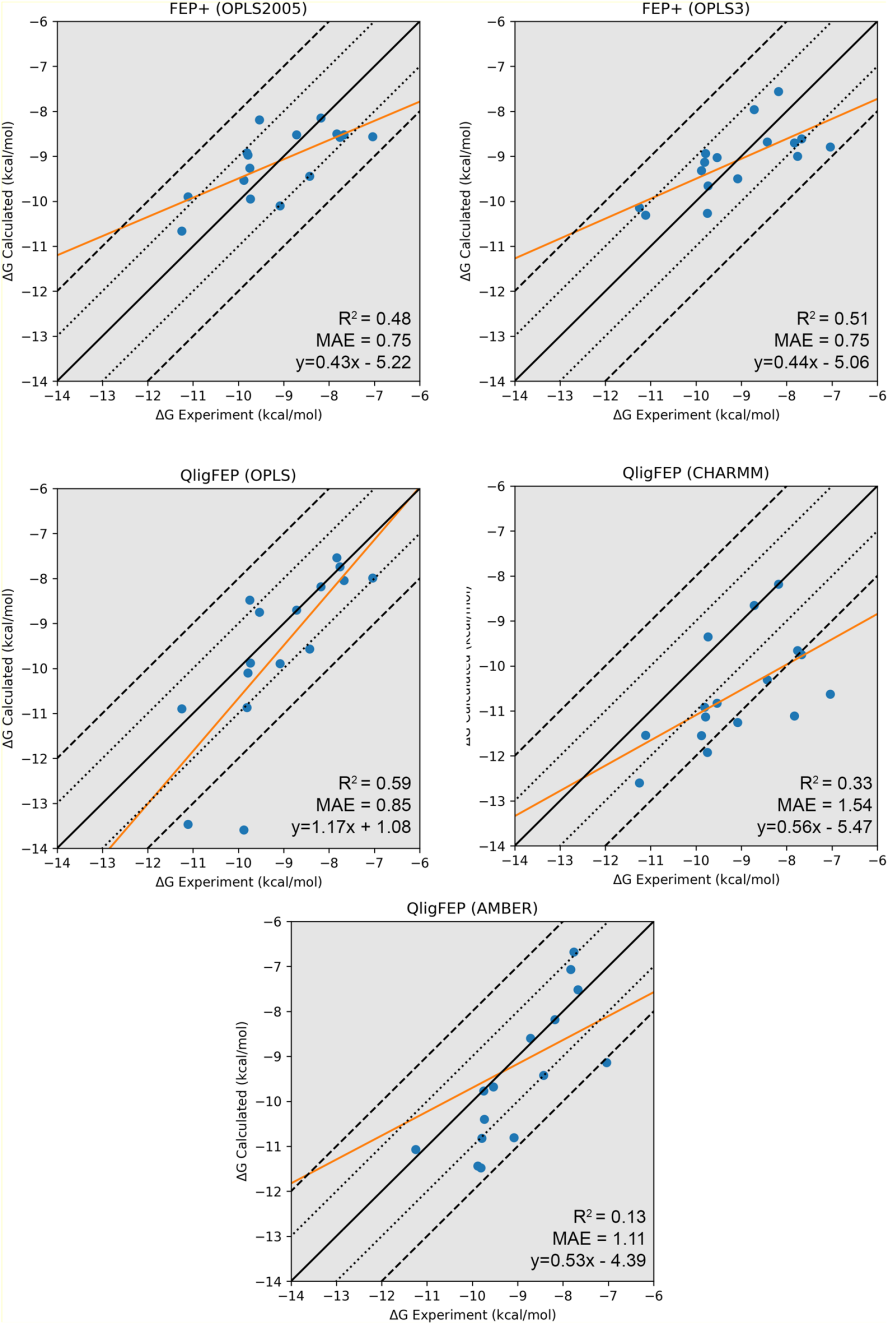
Scatter plots for the calculated and experimental relative binding free energies (∆*G*_bind_, kcal/mol) for the series of 16 CDK2 inhibitors, taking 1h1r as reference. The orange line represents the calculated linear equation for the correlation coefficient R^2^. The black line represents a perfect correlation, and the two dashed lines are +/− 1 and +/− 2 kcal/mol respectively

The first example of ligand binding involves a series of antagonists of the CDK2 receptor [71]. This receptor is a drug target in oncology, and several inhibitors have been identified. Indeed, many solved CDK2—inhibitor crystal structures exist, making this system particularly suitable for testing structure-based computational methods. In an earlier FEP study on this system, Wang et al. [49] examined the relative affinities for a set of 16 inhibitors using FEP+, which included 6 crystallized inhibitor-protein complexes and 10 analogs of these inhibitors, with a manually designed cycle closure and the OPLS2005 force field. Later on, the same dataset was used to test the automated FEP workflow implemented in FEP+, in combination with the new proprietary versions of the OPLS force field [17, 29]. We herein use this dataset to evaluate the performance of QligFEP in a (retrospective) ligand design project. Figure 2 shows the binding mode of the main scaffold (panel A) and the perturbation scheme applied in this study (panel B). The six structures solved in complex with CDK2 are depicted as square nodes, with their PDB codes indicated, while the remainder of the ligands (ellipsoidal boxes) were considered in the docking poses kindly provided by Wang et al. All substituents involved different modifications of the benzene ring of ligand 1h1q, which forms the central node in our FEP strategy. The results are summarized in Table 2 and Fig. 3, which also includes the values obtained with Schrodinger’s FEP+ for comparative purposes [29], together with the relevant statistical figures of merit. The affinity values, computed relative to the reference compound 1h1q, are here transformed to absolute binding free energies by scaling to the affinity of this compound, to facilitate the comparison with the FEP+ results. QligFEP results show very good agreement with the experimental data, in particular with the OPLS force field. The low MAE obtained (0.85 kcal/mol) is very encouraging and only slightly higher than the MAE computed from the FEP+ results. The correlation of QligFEP-OPLS results with experimental data, on the other hand, is the highest of the methods compared. Not only does this model result in the best correlation coefficient, but the statistics actually denote an important improvement in the predictive power and interpretation of this model as compared to all other models in Table 2, with a slope very close to the ideal value of 1 (Fig. 3) and the smallest intercept of 1 kcal/mol. These values are in contrast with the two FEP+ models and the QligFEP models obtained with the AMBER and CHARMM force fields, where the slope is around 0.5 and the intercept close to − 5 kcal/mol, indicating that most of the variability of the data is actually not explained by the models (Fig. 3). We should stress that in QligFEP no attempts have been made to increase the quality of the ligand parameters generated by any of the automated parameterization methods. In particular, when using either GAFF or CGenFF, quite a few critical warnings were ignored, which in a real (prospective) application one would certainly optimize, and this could partially account for the lower performance as compared to the results using the OPLS force field. However, we feel that such an optimization procedure would be beyond the scope of this work, as the aim of the reported tool is to automate FEP calculations, not parameter generation, and the possibility of using different force fields as per the preference (and experience) of the user. In this sense, we would also like to stress that the QligFEP calculations were performed using the OPLS-AA/M model, which is very similar to OPLS2005 but supposedly inferior to the latest version OPLS3, a proprietary version not publicly available. However, as stated before the performance of OPLS3 with a more complex cycle closure strategy only improves slightly in terms of the MAE (0.1 kcal/mol), whilst the R^2^ and interpretation of the model is superior in QligFEP.

**Table 2:** Calculated and experimental relative binding free energies between pairs of the 16 CDK2 inhibitors, corresponding to the FEP pathways depicted in Fig. 2

Ligand	Experiment^a^	Calculated^b^				
		FEP+		QligFEP		
		OPLS2005	OPLS3	OPLS2005	AMBER	CHARMM
1h1q	− 8.18	− 8.15 ± 0.00	− 7.56 ± 0.00	–	–	–
1h1r	− 7.67	− 8.52 ± 0.26	− 8.61 ± 0.17	− 8.04 ± 0.11	− 7.52 ± 0.31	− 9.75 ± 0.45
1h1 s	− 11.25	− 10.66 ± 0.40	− 10.14 ± 0.56	− 10.90 ± 0.28	− 11.07 ± 0.41	− 12.60 ± 0.72
1oi9	− 9.74	− 9.95 ± 0.32	− 9.66 ± 0.22	− 9.88 ± 0.16	− 10.40 ± 0.24	− 9.35 ± 0.49
1oiu	− 9.08	− 10.10 ± 0.24	− 9.50 ± 0.47	− 9.89 ± 0.26	− 10.81 ± 0.74	− 11.26 ± 0.63
1oiy	− 9.79	− 8.97 ± 0.29	− 8.94 ± 0.39	− 10.10 ± 0.23	− 10.82 ± 0.48	− 11.13 ± 0.52
17	− 7.04	− 8.56 ± 0.19	− 8.79 ± 0.13	− 7.99 ± 0.11	− 9.14 ± 0.51	− 10.63 ± 0.33
20	− 8.72	− 8.52 ± 0.33	− 7.96 ± 0.28	− 8.70 ± 0.18	− 8.60 ± 0.23	− 8.65 ± 0.37
21	− 7.83	− 8.50 ± 0.27	− 8.70 ± 0.15	− 7.54 ± 0.15	− 7.07 ± 0.35	− 11.11 ± 0.39
22	− 7.76	− 8.58 ± 0.23	− 9.00 ± 0.18	− 7.74 ± 0.15	− 6.68 ± 0.51	− 9.66 ± 0.47
26	− 8.43	− 9.44 ± 0.33	− 8.68 ± 0.39	− 9.57 ± 0.14	− 9.42 ± 0.44	− 10.31 ± 0.59
28	− 11.11	− 9.90 ± 0.49	− 10.31 ± 0.49	− 13.47 ± 1.10	− 5.45 ± 0.93	− 11.54 ± 0.89
29	− 9.88	− 9.53 ± 0.47	− 9.32 ± 0.61	− 13.59 ± 0.99	− 11.44 ± 1.18	− 11.55 ± 1.21
30	− 9.81	− 8.92 ± 0.38	− 9.13 ± 0.48	− 10.87 ± 0.60	− 11.48 ± 0.77	− 10.92 ± 0.50
31	− 9.54	− 8.19 ± 0.41	− 9.03 ± 0.46	− 8.75 ± 0.19	− 9.68 ± 0.48	− 10.83 ± 0.91
32	− 9.75	− 9.26 ± 0.47	− 10.27 ± 0.49	− 8.48 ± 0.84	− 9.77 ± 0.65	− 11.92 ± 0.93
R^2^ [95% CI]		0.48 [0.09–0.78]	0.51 [0.11–0.79]	0.59 [0.20–0.84]	0.13 [0.01–0.52]	0.33 [0.01–0.69]
MAE		0.75 (0.69^c^)	0.75 (0.79^c^)	0.85	1.11	1.54
Slope		0.43	0.44	1.17	0.53	0.56
Intercept		− 5.22	− 5.06	1.08	− 4.39	− 5.47

^a^Experimental ∆*IC*_50_ values extracted from Ref. [71] and are transformed into ∆∆*G*_bind_ using the relation $\Delta \Delta G_{bind,exp}^{o} = RTln\left( {\frac{{IC_{50} \left( B \right)}}{{IC_{50} \left( A \right)}}} \right)$

^b^All energies are in kcal/mol, with standard error of the mean (SEM) estimated from replicate simulations for Q, and cycle closure errors for FEP+ [29]

^c^MAE taken from Ref. [29], based on cycle closure correction

### A_2A_AR antagonist binding

**Fig. 4:**
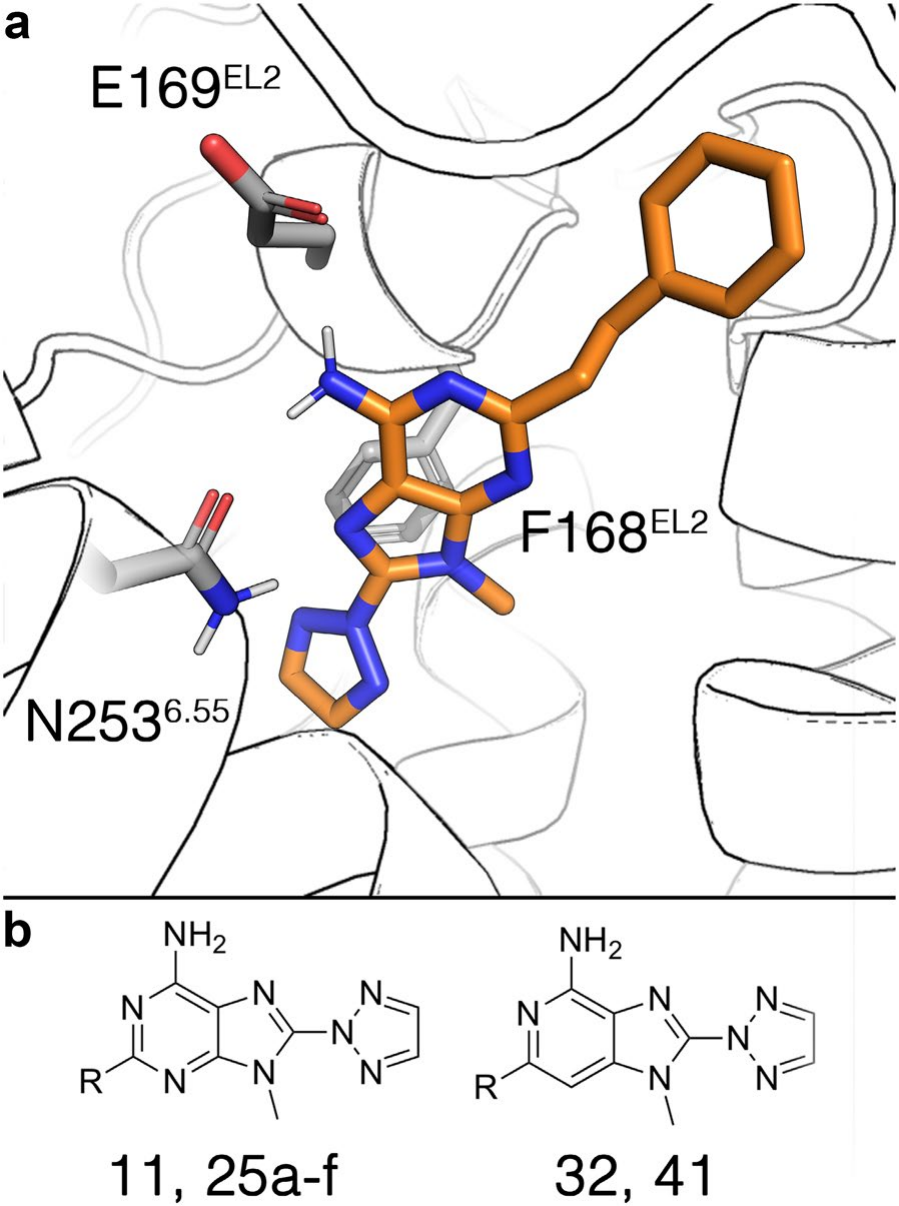
**a** Binding mode of compound 25d to the A_2A_AR, with the key interacting residues denoted by sticks. **b** Core scaffolds of the two series investigated in this work

The A_2A_AR has been widely used by us and others as a test-case for the performance of computational methods in the design of ligands for more complex membrane systems [72]. The advantage of using spherical boundary conditions becomes evident in these systems, where a sphere centered on the binding site with a diameter of 50 Å contains approximately 7.400 atoms (see Fig. 1). This significantly decreases the computational time needed for a simulation as compared to a periodic system of i.e. an optimized hexagonal shaped box with ~ 42.000 atoms. We have shown that calculations performed on larger sphere systems of the same A_2A_AR receptor complex result in larger errors, but yield the same average changes in binding free energies [12, 73]. On the related A_3_AR, our FEP approach now automated under QligFEP was used to elucidate the role of a nitrogen substitution in the core of antagonists with a pyrimidine scaffold [74]. Here, we apply QligFEP to compute the relative affinities of 8 analogs of the triazol-2-yl-9 H-purin-6-ylamine (ST1535, Fig. 4) [31]. The binding mode of this compound, shown in Fig. 4, was inferred by analogy to the binding mode of the triazolopyrimidine antagonist ZM241385 [75]. This includes key hydrogen bonds with N253^6.55^ and E169^EL2^ (subscripts indicate the general GPCR topological nomenclature [31]) and π–π stacking interactions between the bicyclic core of the ligand and F168^EL2^, which are common interactions in adenosine receptor ligand recognition [76]. Table 3 reports the calculated relative free energy (∆∆*G*_bind_) of each compound to the simplest ligand compound **11**. For comparison, the experimental values are shown together with the values obtained by Lenselink et al. [30] using FEP+ under PBC. In a first attempt, each of the eight analogs was directly perturbed to the reference ligand **11** (note the nomenclature of the compounds following the original report by Minetti et al. [31]). Six out of the eight perturbations are well converged, whereas we observed a large SEM (over 1.6 kcal/mol) for the perturbations including the largest compounds **25c** and **25d**. This provides some insight in the convergence limitations of a direct perturbation between two related ligands. In this case, it was evidenced that the phenylethyl (**25d**) or pentyl (**25c**) substitutions are too large to compute reliable relative affinities when directly perturbed to hydrogen (**11**). To solve this, we created an alternative pathway that involved a smoother annihilation scheme through two intermediate steps linking these ligands to the reference compound **11**, i.e. **25d** → **25c** → **25b** → **11**. The calculated relative affinities with QligFEP show excellent agreement with the experimental data (Table 3). Moreover, the statistical figures of merit (R^2^ = 0.88; MAE = 0.72 kcal/mol) are equivalent to those reported by Lenselink et al. (R^2^ = 0.78; MAE = 0.68 kcal/mol). Notably, in that work the proprietary OPLS3 force field was used and the level of performance indicated was only achieved after applying the cycle-closure strategy, which includes a total of 17 perturbations in redundant cycles and the transformation of the data to include ∆G values relative to **11**. If only the perturbations to **11** are selected and the relative binding free energies are used (which give a shorter span and thereafter are more sensible to fluctuations in the overall correlation), the correlation of the FEP+ strategy is moderately hampered (R^2^ = 0.50, as opposed to the corresponding value of R^2^ = 0.74 for QligFEP). It is worth noting that two perturbations that we accurately model include changes in the core ring (**32** and **41**–**11**), which belong to the scaffold hopping modifications that we discuss in more detail below.

**Table 3:** Calculated and experimental shifts in binding free energies of a series of A_2A_AR antagonists, relative to the parent compound **11**

Compound	R	Binding free energies (ΔΔ*G*_bind_, kcal/mol)		
		Experiment	FEP+^a^	QLigFEP
11	H	–	–	–
25a	CH_3_	0.25	0.53 ± 0.04	0.74 ± 0.34
25b	(CH_2_)_3_CH_3_	− 1.15	− 0.34 ± 0.10	− 0.96 ± 0.81
25c	(CH_2_)_4_CH_3_	− 1.56	− 0.56 ± 0.09	− 0.93 ± 0.86
25d	(CH_2_)_2_Ph	− 1.35	− 0.69 ± 0.11	− 0.75 ± 0.95
25e	CH(CH_4_)_2_	1.39	0.88 ± 0.09	2.46 ± 0.99
25f	(CH2)_2_CH_3_	0.35	0.23 ± 0.07	2.17 ± 1.07
32	H	0.06	1.24 ± 0.18	0.67 ± 0.22
41	CH_3_	0.56	3.08 ± 0.16	0.98 ± 0.33
R^2^ [95% CI]			0.49 (0.78^a^) [0.00–0.88]	0.74 (0.88^b^) [0.16–0.95]
MAE			0.74 (0.68^a^)	0.72
Slope			0.74	0.99
Intercept			0.56	0.49

^a^MAE reported in the original work form Lenselink et al. [30], based on cycle closure correction and using the affinity of **11** to calculate ∆G values

^b^R^2^ for ∆G values using the same strategy of using ∆G with **11** as a reference

### Scaffold hopping

**Fig. 5:**
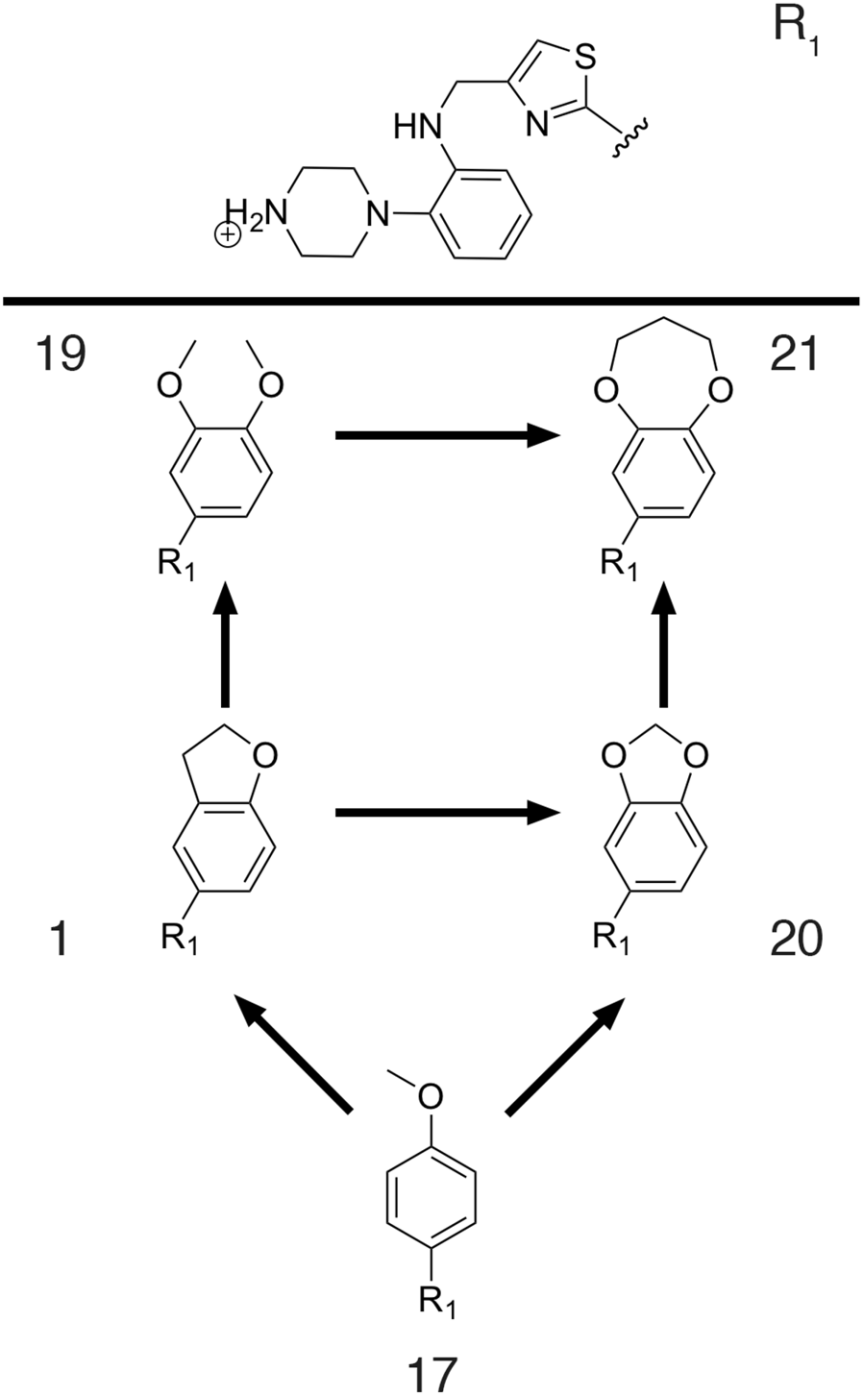
Core of the scaffold of the Chk1 inhibitor series, showing the variable position explored by scaffold hopping with the five different R-group modifications (bottom). The pathways chosen to connect these groups through FEP simulations to calculate the relative change in binding free energies are indicated by arrows, the corresponding values reported in Table 4

Thus far, examples presented in this work involved typical lead-optimization strategies, where R-group substituents are introduced to a scaffold with the aim to increase binding affinity. Another strategy within the medicinal chemist’s toolbox is to change the core of the lead ligand while retaining peripheral R-group substituents that are identified as crucial for protein–ligand recognition [59, 77]. Such a procedure, also referred to as ‘scaffold-hopping’, can be extremely useful to overcome problems related to the initial scaffold, such as ADMET properties or reactivity, as well as to expand the chemical space and overcome patentability problems [77]. However, scaffold hopping has been elusive to FEP approaches as it often involves significant amounts of changes in the bond topologies within the ligand series. Within a single topology framework, one would have to make or break bonds to include such changes, something that is prone to numerical instabilities due to the asymptotic nature of harmonic potentials representing bond stretching/closing in classical force fields [21, 78]. One strategy to overcome this is to change the asymptotic nature of the bond by introducing a softcore bond potential [78]. Within the QligFEP dual-topology framework, we introduce an alternative approach, since no bonded terms are altered within the transition of the ligand pair, even if the bond topology changes. Additionally, since the two molecules exist as two unrelated entities, the necessary dummy atoms do not experience any strain from residual ‘real’ atoms, and the thermodynamic cycle can be formally closed [21]. To test the applicability of this approach in a typical scaffold-hopping case, we performed a total of six perturbations between five inhibitors of the Checkpoint kinase 1 (Chk1, see Fig. 5). This set was extracted from a recent benchmark on scaffold hopping using FEP+ applying the aforementioned softcore potentials, and it is illustrative of the various topological changes observed in these type of modifications, i.e. ring opening, ring closure, changing the ring size and the substitution of atoms within a ring. The results are summarized in Table 4. Even if the changes in affinity within the series are relatively small (which is typically the case in a successful scaffold hoping project, i.e. to discover new chemical entities with similar binding affinities) these are very successfully captured with our QligFEP approach, with an MAE of 0.32 kcal/mol. More importantly, with the only exception of the pair 19 → 21 involving a ring formation, see Fig. 5, the simulations are well converged with a SEM typically under 0.5 kcal/mol (Table 4). Notably, these values are quite comparable with those reported with the alternative FEP+ approach [78], even though a different force field version was used (OPLS2005 in our methods versus OPLS3 in FEP+, see Table 4). This shows that the practical advantages of a dual topology approach in scaffold hopping do not cause a lower performance as compared to single topology approaches.

**Table 4:** Calculated and experimental relative binding free energies between pairs of Chk1 inhibitors, as indicated in Fig. 5

Ligand pair	Binding free energies [ΔΔG (kcal/mol)]		
	Experiment	FEP+	QligFEP
17 → 1	0.02	− 0.85 ± 0.09	− 0.39 ± 0.41
1 → 19	1.15	0.93 ± 0.08	0.41 ± 0.48
1 → 20	0.49	0.60 ± 0.03	0.30 ± 0.30
17 → 20	0.51	0.06 ± 0.10	0.32 ± 0.56
19 → 21	− 0.59	0.03 ± 0.15	− 0.14 ± 1.17
20 → 21	0.07	0.08 ± 0.16	− 0.15 ± 0.27
R^2^ [95% CI]		0.61 [0.00–0.95]	0.73 [0.02–0.97]
MAE		0.37	0.32
Slope		1.40	1.58
intercept		0.19	0.05

## Discussion

We present QligFEP, an automated workflow to setup, run and analyze ligand free energy perturbation calculations within a structure-based drug design framework. The workflow is built as an API that interacts with the open-source software package Q. It aims to automate the tedious process of creating input and parameters files and to facilitate a robust setup to perform free energy calculations between a given pair of ligands to calculate e.g. their relative binding affinities. We show how this approach can be scaled up to perform pair comparisons in a high-throughput fashion for a versatile set of ligands and receptors, which to the best of our knowledge is achievable with a limited amount of software packages [9, 29, 49].

Given a dataset with a number *l* of ligands, one can eventually perform all possible pair comparisons, which is proportional to *l*^*2*^. This brings up the question on how to optimize the design of the FEP strategy such that a selected sample of pair comparisons (l_i_, l_j_) represent the spectrum of relative affinities to cover the whole dataset whilst minimizing the structural changes involved in order to ensure sufficient convergence. One way to approach this problem is through the use of MCS algorithms, further combined with the idea of cycle closures, to validate the FEP pathways (i.e., the addition to the ∆∆*G*_bind_ calculated along a given path A → B → C → A should be zero). While for big datasets this automated approach is a useful solution, in most cases one can design pathways based on basic medicinal chemistry knowledge, and assess the feasibility of this design solely based on the convergence achieved. This is nicely illustrated in this work by the calculations performed on the A_2A_AR antagonists set. The initial design of the FEP pathway involved the calculation of the affinities of each compound relative to a single reference compound. This is, in our opinion, the recommended approach in a lead optimization project, where typically one only knows the affinity of a reference compound and variations of one or several substituents are designed. In this case, two perturbations showed low convergence, detected by an abnormally high SEM within the replicate simulations of the system. We redesigned the strategy to circumvent this issue, involving a cycle closure defined by four compounds within the dataset. This case also illustrates the increased computational efficiency of QligFEP as compared to the FEP+ methodology. First, the cycle-closure strategy used in FEP+ involved 17 perturbations as compared to the 10 perturbations in our FEP pathway. Second, our system setup with SBC is approximately 6 times smaller than the PBC setup used by FEP+. Third, the simulation times reported are 10 times longer (see methods section) for a single simulation per ligand pair. Taken all together, QligFEP provides an alternative for efficient FEP simulations, at a substantial reduction of the computational cost as compared with existing commercial software. In other words, we show an alternative to a more exhaustive cycle closure, which is based on starting with a simple FEP design based on the MCS idea, detect potential problematic pairs based on convergence using the SEM as a metric and iterative processing of the obtained data if needed.

Another advantage of our approach is the implementation of a dual topology representation, which allows the comparison of molecules with unrelated topologies. This is a particularly interesting approach in, for instance, scaffold hopping where one wants to normally compare different chemical scaffolds. In a single topology framework, where a one-to-one mapping of atoms between the two states is used, this potentially includes the transformation of bonded terms to represent the change in bond topology. In contrast, with QligFEP this problem can be circumvented, whilst still achieving excellent correlation with experiment. Our initial results are promising, and the next direction in our research will be to apply the QligFEP dual topology approach the binding affinity prediction of fragment-like compounds, which involves comparing different chemical scaffolds where even potential multiple binding modes can be assessed.

## Conclusion

QligFEP provides a versatile, robust and accurate framework to routinely perform FEP calculations in structure-based ligand design projects. We herein show the performance on a diverse benchmark set of systems and ligands, showing agreement between experiment and calculations similar to that of other state-of-the-art methods. QligFEP is implemented as an easy to use, modular API, which interacts with the open-source MD engine Q. Additionally, our package already works with a number of standard force fields, whilst the implementation of additional force fields is currently under development. The benchmark test sets included in this study are part of the tutorials and provide a useful start for users with limited training in free energy calculations. QligFEP and its associate MD engine Q are available free of charge through github.

## Abbreviations

BAR: Bennet’s acceptance ratio
CDK2: Cyclin-dependent Kinase 2
FEP: free energy perturbation
GPCR: G protein-coupled receptor
MAE: mean unassigned error
MD: molecular dynamics
PBC: periodic boundary conditions
SBC: spherical boundary conditions
SEM: standard error of the mean
